# Construction of a Corneal Stromal Equivalent with SMILE-Derived Lenticules and Fibrin Glue

**DOI:** 10.1038/srep33848

**Published:** 2016-09-21

**Authors:** Houfa Yin, Peijin Qiu, Fang Wu, Wei Zhang, Wenqi Teng, Zhenwei Qin, Chao Li, Jiaojie Zhou, Zhi Fang, Qiaomei Tang, Qiuli Fu, Jian Ma, Yabo Yang

**Affiliations:** 1 Eye Center, Second Affiliated Hospital, School of Medicine, Zhejiang University, Hangzhou, China; 2 Department of Orthopedics, Second Affiliated Hospital, School of Medicine, Zhejiang University, Hangzhou, China; 3 Department of Surgery, Second Affiliated Hospital, School of Medicine, Zhejiang University, Hangzhou, China

## Abstract

The scarcity of corneal tissue to treat deep corneal defects and corneal perforations remains a challenge. Currently, small incision lenticule extraction (SMILE)-derived lenticules appear to be a promising alternative for the treatment of these conditions. However, the thickness and toughness of a single piece of lenticule are limited. To overcome these limitations, we constructed a corneal stromal equivalent with SMILE-derived lenticules and fibrin glue. *In vitro* cell culture revealed that the corneal stromal equivalent could provide a suitable scaffold for the survival and proliferation of corneal epithelial cells, which formed a continuous pluristratified epithelium with the expression of characteristic markers. Finally, anterior lamellar keratoplasty in rabbits demonstrated that the corneal stromal equivalent with decellularized lenticules and fibrin glue could repair the anterior region of the stroma, leading to re-epithelialization and recovery of both transparency and ultrastructural organization. Corneal neovascularization, graft degradation, and corneal rejection were not observed within 3 months. Taken together, the corneal stromal equivalent with SMILE-derived lenticules and fibrin glue appears to be a safe and effective alternative for the repair of damage to the anterior cornea, which may provide new avenues in the treatment of deep corneal defects or corneal perforations.

Deep corneal defects and corneal perforations can result from various infectious and noninfectious disorders, including microbial keratitis, trauma, degeneration, and immune disorders^1^,^2^. These serious conditions may result in devastating visual consequences and must be managed by immediate treatment to preserve the anatomic integrity of the cornea and prevent complications such as endophthalmitis, secondary glaucoma, and subsequent permanent vision loss^1^. However, management of deep corneal defects and corneal perforations, especially those secondary to underlying autoimmune disease, remains a challenge for ophthalmic surgeons^3^.

The treatment options for these cases include tissue adhesives, conjunctival flap, patching with corneal tissue, and amniotic membrane graft^1^. However, there are limitations in their application. A conjunctival flap procedure is a traumatic technique, as it draws new vessels to the cornea and destroys conjunctival tissue^4^. The amniotic membrane is an uneven biological tissue that may harbor biological hazards and is not universally available^4^,^5^. Moreover, tissue adhesives, such as cyanoacrylate and fibrin adhesive, and amniotic membrane transplantation are not always effective, particularly when perforations are larger than 3 mm in diameter^6^. Corneal transplantation is a definitive treatment; however, a shortage of donor corneas is a major constraint, especially in developing countries, where the need is highest^7^,^8^,^9^. Furthermore, the underlying active inflammatory condition may limit the success rate of corneal transplantation^1^,^10^.

Lenticules, which are extracted during the small incision lenticule extraction (SMILE) procedure for myopic correction, are increasingly being studied in animal implantation testing and preclinical/clinical applications, including intrastromal lenticule implantation for the treatment of aphakia, hypermetropia, keratoconus, and presbyopia^11^,^12^,^13^,^14^,^15^,^16^. More recently, we used multilayer SMILE-derived lenticules to treat corneal perforations^17^. Subsequently, Bhandari *et al*. also reported the efficacy of SMILE-derived lenticule patch grafts for the management of corneal microperforations and lamellar corneal defects^18^. These studies expanded the current strategies for the management of corneal perforation and partial-thickness corneal defects and demonstrated that the use of SMILE-derived lenticules is a safe, effective, easily accessible, and inexpensive alternative for corneal stroma reconstruction. However, due to the minor thickness (most less than 160 μm) and limited toughness of a single piece of lenticule, the manipulation of separate pieces of lenticule is difficult and time-consuming. In addition, overlapping lenticules may be misaligned or loose-fitting, which may lead to instability of the wound structure and delay of corneal epithelial wound healing^17^. Furthermore, no histological and ultrastructural studies on this constructed corneal stromal equivalent have been reported.

In the present study, we constructed a corneal stromal equivalent with SMILE-derived lenticules and fibrin glue. Then, we utilized *in vitro* cell culture to assess the effect of this constructed corneal stromal equivalent on the proliferation, phenotype, and adhesion of corneal epithelial cells. We also determined the suitability of using the corneal stromal equivalent with decellularized lenticules and fibrin glue for corneal stroma reconstruction *in vivo*.

## Materials and Methods

The use of human tissue samples was approved by the ethics committee of the Second Affiliated Hospital, School of Medicine, Zhejiang University, and the procedures used conformed to the tenets of the Declaration of Helsinki. Informed consent was obtained from all patients. Male New Zealand white rabbits (weighing 2~2.5 kg, aging 3~4 months) were supplied by the Academy of Medical Sciences of Zhejiang province. All animal experiments were approved by the Animal Ethics Committee of the Second Affiliated Hospital, School of Medicine, Zhejiang University and were in accordance with the National Institutes of Health Guide for the Care and Use of Animals and the Association for Research in Vision and Ophthalmology (ARVO) statement for the use of animals in ophthalmic and vision research.

### Construction of corneal stromal equivalent

**Figure 1:**
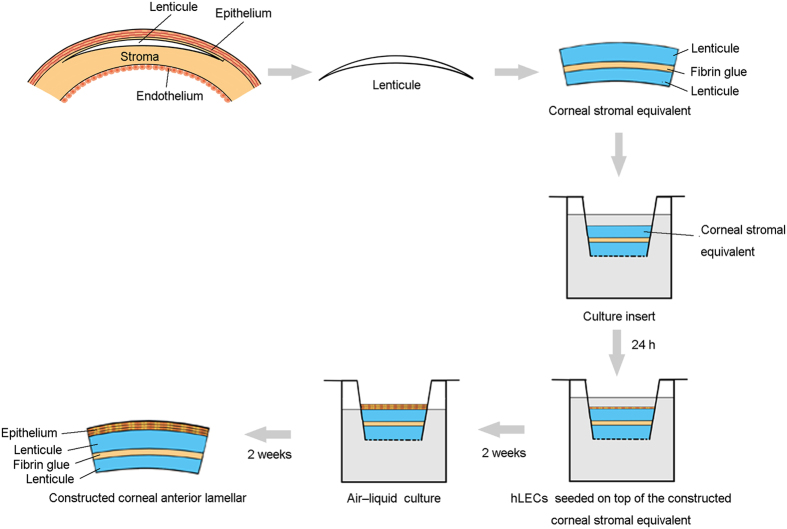
Serial construction of the corneal anterior lamellar. First, the lenticule is extracted from the human cornea using the VisuMax Femtosecond Laser System. Then, 2 lenticules derived from the same donor are adhered to each other by fibrin glue and soaked in culture medium for 24 h. Finally, human limbal epithelial cells are seeded on top of the constructed corneal stromal equivalent. The air-liquid culture technique is used to induce epithelial stratification.

Human corneal lenticules were extracted during SMILE procedures using the VisuMax Femtosecond Laser System (Carl Zeiss Meditec AG, Jena, Germany) as previously described^17^. Only lenticules with a diameter of 6.6 mm and a central thickness of ≥100 μm were selected for the construction of corneal stromal equivalents. A 10-0 nylon suture was applied with the knot tied on the superior aspect of the lenticule to determine the correct side at the time of the subsequent procedure. The lenticule was immediately transferred to a sterile Eppendorf tube that was pre-filled with Dulbecco’s modified Eagle’s medium/F12 (DMEM/F12; Gibco, NY, USA) containing 100 U/ml penicillin G, 100 μg/ml streptomycin sulfate, and 1 μg/ml amphoptericin B (Invitrogen, Carlsbad, USA) and transported at 4 °C to the laboratory. The lenticule was rinsed 3 times with phosphate-buffered saline (PBS). For construction of the corneal stromal equivalent, 2 lenticules derived from the same donor were adhered to each other by fibrin glue (Shanghai Raas Blood Products Co., Ltd, Shanghai, China) with the superior aspect upward (Fig. 1).

### Cell culture on corneal stromal equivalent

Human corneoscleral rims were obtained from the Eye Center, Second Affiliated Hospital, School of Medicine, Zhejiang University after the removal of the central corneal buttons for corneal transplantation. Human limbal epithelial cells (hLECs) were cultured as previously described with necessary modifications^19^. To isolate hLECs, limbal segments were digested at 37 °C for 1 h in DMEM/F-12 containing 2 mg/mL dispase II (Invitrogen). Then, the loose limbal epithelial sheets were peeled off and digested in 0.25% trypsin-EDTA (Invitrogen) at 37 °C for 30 min. Cells were washed and re-suspended in culture medium. To ensure both submerged culture and differentiation of the multilayered epithelium by growth at the air-liquid interface, the constructed corneal stromal equivalent was placed in a cell culture insert (Transwell, Corning, NY, USA) with 0.4 μm pore size and 6.5 mm diameter^20^. The constructed equivalent was soaked in culture medium at 37 °C for 24 h before cell seeding. Then, hLECs were seeded on top of the constructed corneal stromal equivalent (500,000 cells per equivalent) and cultured for 2 weeks submerged in culture medium. To induce stratification, the air-liquid culture technique was used for 2 more weeks (Fig. 1). In all cases, the culture medium was DMEM/F-12 supplemented with 10% fetal bovine serum (FBS, Gibco), 1% human corneal growth supplement (HCGS, Gibco), 5 ng/ml epidermal growth factor (Sigma-Aldrich), 100 U/ml penicillin G, 100 μg/ml streptomycin sulfate, 1 μg/ml amphoptericin B (Invitrogen), and 163 μg/ml aprotinin (Sigma-Aldrich). The medium was changed every 2-3 days.

### Decellularization of SMILE-derived lenticules

**Figure 2:**
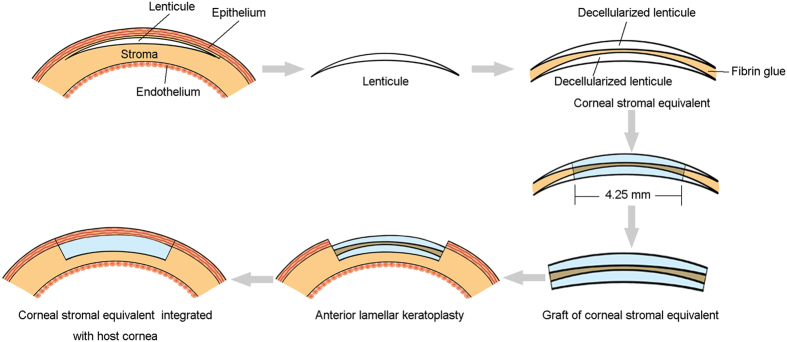
Diagram of the steps of the implantation of the decellularized corneal stromal equivalent in rabbits. First, the lenticule is extracted from the human cornea using the VisuMax Femtosecond Laser System. Then, 2 decellularized lenticules derived from the same donor are adhered to each other by fibrin glue. Finally, the graft of the corneal stromal equivalent (4.25 mm in diameter) is implanted into the cornea of a rabbit by anterior lamellar keratoplasty.

Lenticules with a diameter of 6.6 mm and central thickness of ≥100 μm were selected, and the superior aspect of the lenticules was labelled as described above. Decellularization of lenticules was performed as previously described^19^. Briefly, lenticules were washed 3 times in PBS and then incubated in 1.5 M sodium chloride (NaCl) solution for 48 h with NaCl change after 24 h. Subsequently, the lenticules were treated with 5 U/ml DNAse and 5 U/mL RNAse (Sigma-Aldrich) for 48 h. Next, lenticules were washed with PBS for 72 h with PBS changed every 24 h. The decellularization procedure was performed at room temperature under continuous agitation. For anterior lamellar keratoplasty, 2 decellularized lenticules derived from the same donor were adhered to each other by fibrin glue (Shanghai Raas Blood Products Co., Ltd) with the superior aspect upward and dehydrated in glycerol to the normal thickness (Fig. 2).

### Anterior lamellar keratoplasty (ALK) in rabbits

**Figure 3:**
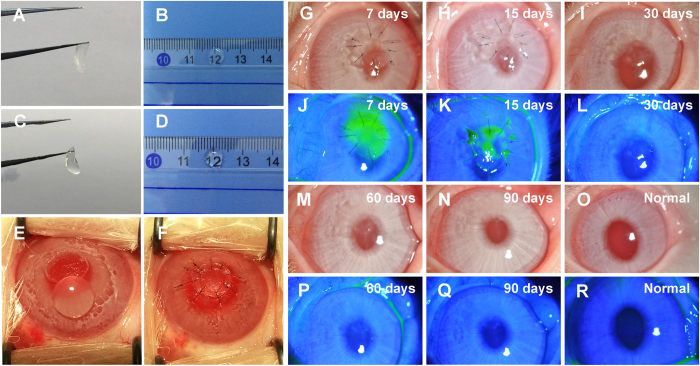
General appearance of decellularized lenticules and ALK in rabbits. (**A,B**) The lenticule was opaque due to the decellularization process. (**C,D**) The decellularized lenticule became transparent after deturgescence with glycerol. (**E,F**) Surgical steps of ALK. (**G–L–N,P,Q**) Representative slit-lamp photographs and sodium fluorescein staining of rabbit eyes obtained 7, 15, 30, 60, and 90 days after ALK. (**O,R**) Slit-lamp photographs and sodium fluorescein staining of normal rabbit eyes.

Twelve corneal stromal equivalents composed of 2 decellularized lenticules and fibrin glue (4.25 mm diameter) were implanted into the right corneas (transplanted group) of 12 rabbits by ALK. Contralateral unoperated corneas served as the control group. Briefly, the rabbits were anesthetized with an intravenous injection of a mixture of 20 mg/ml ketamine and 2 mg/ml xylazine diluted in saline. To perform ALK, a circular incision with a depth of approximately 250 μm and a diameter of 4 mm was made in the right eye. The anterior lamellar stroma was then dissected using an operating knife. The corneal stromal equivalent graft was sutured into the recipient bed with interrupted 10-0 sutures (Figs 2 and 3E,F). Tobramycin and dexamethasone eye drops were used 4 times daily for 2 weeks after ALK. Following surgery, rabbits were examined using slit-lamp biomicroscopy with sodium fluorescein staining to assess epithelial integrity, corneal optical clarity, neovascularization, and the state of the grafts. In addition, corneal topography evaluation using Pentacam (Oculus, Wetzlar, Germany) and anterior segment optical coherence tomography (AS-OCT) using Visante (Carl Zeiss Meditec, Dublin, CA, USA) were performed to assess corneal deformation and corneal curvature. Four rabbits were euthanized at 0.5, 2, and 3 months post-surgery, and the corneal specimens were prepared for hematoxylin-eosin (H&E) staining and transmission electron microscopy (TEM).

### H&E and immunofluorescence staining

For H&E staining, tissues were fixed in 4% paraformaldehyde (PFA), and embedded in paraffin. Three μm sections were stained with H&E, and viewed under a light microscope. For immunofluorescence staining, the sections were treated with 0.3% H_2_O_2_ in PBS after deparaffinization and microwave treated antigen retrieval^21^. After 3 rinses with PBS for 5 min each, the sections were incubated with 5% donkey serum to block the non-specific sites. Then, the samples were incubated with anti-CK12 (1:50, Santa Cruz, CA, USA), anti-ABCG2 (1:50, Santa Cruz), or anti-p63α (1:800, Cell Signaling Technology, MA, USA) antibody at 4 °C overnight. The samples were washed 3 times with PBS and incubated with AlexaFluor 488-conjugated IgG (1:300, Invitrogen) or AlexaFluor 555-conjugated IgG (1:300, Invitrogen) for 2 h at room temperature. After washing, sections were counterstained with 4′-6-diamidino-2-phenylindole (DAPI) (Vector, Burlingame, USA), mounted, and examined with a fluorescence microscope.

### Electron microscopy for ultrastructure assessment

Tissues were fixed with 2.5% glutaraldehyde in 0.1 M phosphate buffer (pH 7.4), postfixed with osmium tetroxide in 0.1 M phosphate buffer, and prepared for scanning electron microscopy (SEM) or TEM as previously reported^22^,^23^. The ultrastructure of the specimens was examined and photographed by SEM (SU8010, Hitachi, Japan) or TEM (H-7650, Hitachi, Japan).

## Results

### Characterization of the constructed corneal anterior lamellar

**Figure 4:**
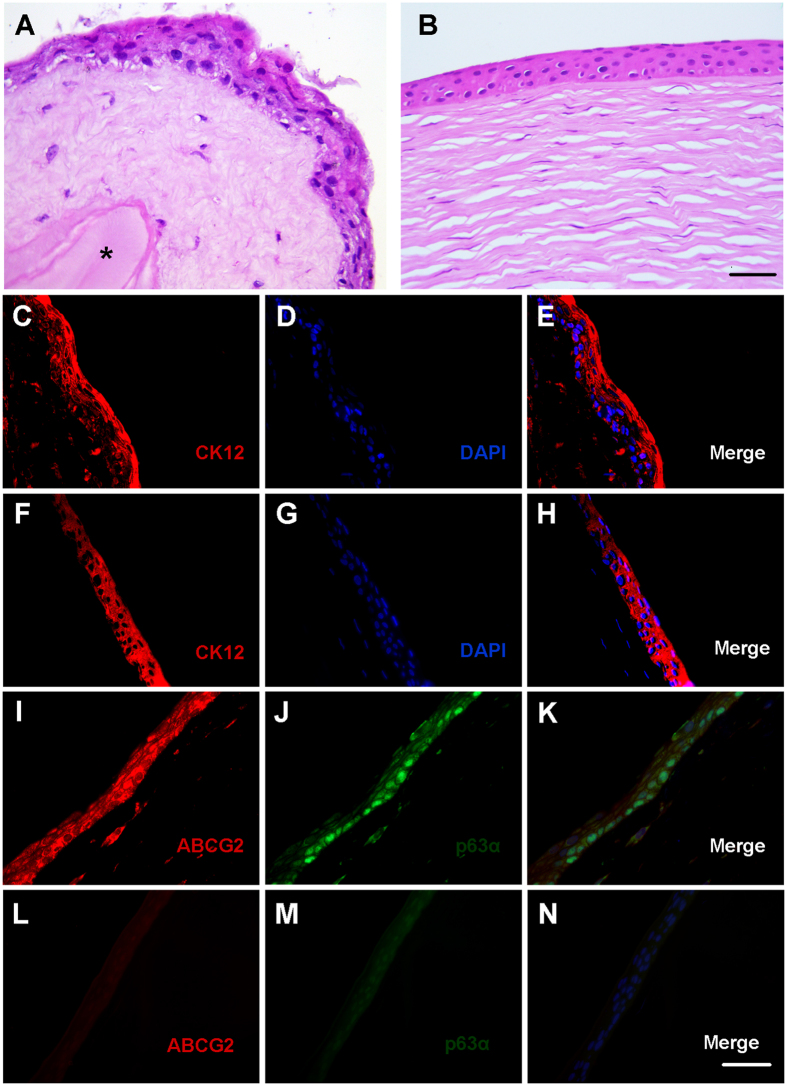
H&E and immunofluorescence staining of the constructed corneal anterior lamellar and native human central cornea. (**A**) Epithelial cells forming a tight, stratified cell layer on top of the corneal stromal equivalent, which contains fresh lenticules and fibrin glue (asterisk). (**B**) Native human central cornea with stratified epithelium attached to cellular stroma. (**C–E**) Corneal specific CK12 expression was noted in all the superficial epithelial cells in the constructed corneal anterior lamellar. (**F–H**) In the native human central cornea, CK12 was strongly expressed in all layers of the central corneal epithelium. (**I–K**) Stem/progenitor cell markers ABCG2 and p63α were expressed in the basal cell layer in the constructed corneal anterior lamellar. (**L–N**) In the native human central cornea, ABCG2 and p63α were absent in the central corneal epithelium. Bar: 20 μm.

**Figure 5:**
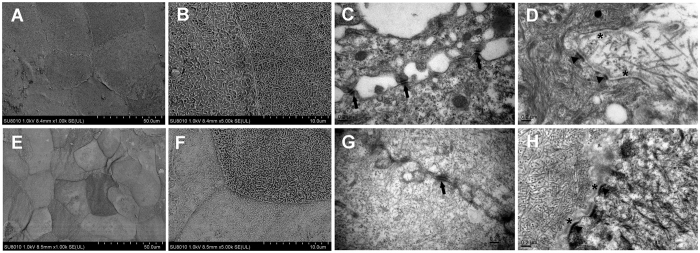
SEM and TEM micrographs of the constructed corneal anterior lamellar and native human cornea. (**A,E**) SEM image revealing characteristic cobblestone-like cells with distinct junctions on the surface of the constructed corneal anterior lamellar (**A**) and native human cornea (**E**). (**B,F**) Higher-magnification versions of (**A**) and (**E**). (**C,G**) TEM image revealing desmosomal junctions (arrows) between adjacent epithelial cells in the constructed corneal anterior lamellar (**C**) and native human cornea (**G**). (**D**) TEM image revealing hemi-desmosomes (arrowheads) connecting the epithelium to the underlying newly synthesized basement membrane (asterisks) in the constructed corneal anterior lamellar. (**H**) TEM image depicting hemi-desmosomes (arrowheads) and basement membrane (asterisks) in native human cornea.

The ability of the constructed corneal stromal equivalent to support corneal epithelial cell growth was evaluated *in vitro*. After air-liquid culture, hLECs formed a continuous pluristratified epithelium consisting of a basal layer of cuboid-shaped cells and 3 to 4 suprabasal layers of elongated cells similar to the native human corneal epithelium (Fig. 4A,B). Fibrin glue remained between the lenticules when cultured with aprotinin, which prevented fibrin degradation (Fig. 4A). By immunolabeling, CK12 was strongly expressed in cells within superficial cell layers (Fig. 4C–E), whereas expression of p63α and ABCG2 was mainly located in the basal cell layer, indicating that the corneal stromal equivalent supported a “limbal” (undifferentiated) phenotype in the basal layer (Fig. 4I–K). In the native human cornea, CK12 was strongly expressed in all layers of the central corneal epithelium, whereas expression of p63α and ABCG2 was absent in the central corneal epithelium (Fig. 4F–H, L–N). SEM and TEM examination of the regenerated corneal epithelial cells on the corneal stromal equivalent revealed similarities to the native human corneal epithelium (Fig. 5). By SEM, the surface of the epithelium on the corneal stromal equivalent was found to exhibit a characteristic cobblestone appearance, which was similar to that of the native human corneal epithelium (Fig. 5A,B,E,F). By TEM, numerous desmosomal junctions were observed between adjacent epithelial cells (Fig. 5C,G). Moreover, basal cells were well attached to the newly synthesized basement membrane and stroma by hemi-desmosomes (Fig. 5D,H). These findings revealed that the constructed corneal stromal equivalent could provide a suitable scaffold for the survival and proliferation of corneal epithelial cells.

### Structure of decellularized lenticules

**Figure 6:**
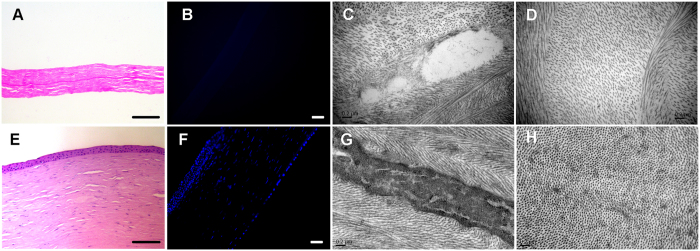
Characteristics of the decellularized lenticule. (**A,B**) H&E staining (**A**) and DAPI staining (**B**) revealing complete removal of cellular components with preservation of fibrillar structures in the decellularized lenticule. Bar: 50 μm. (**E,F**) H&E staining (**E**) and DAPI staining (**F**) of a native human cornea. Bar: 50 μm. (**C,D**) TEM image depicting empty cell space and regular collagen fibrils after the decellularization process, although increased collagen fibril spacing was observed. (**G,H**) TEM image depicting the ultrastructure of the native human cornea.

Although the decellularization process generally caused significant swelling of the corneal tissue, it quickly became transparent after deturgescence with glycerol (Fig. 3A–D), indicating an absence of gross change in the lamellar organization. H&E and DAPI staining revealed complete removal of cellular components while preserving fibrillar structures (Fig. 6A,B,E,F). Additionally, TEM images demonstrated that no cellular debris remained and that the collagen fibrils of the decellularized lenticules were regular, with increased collagen fibril spacing due to swelling (Fig. 6C,D,G,H).

### Implantation of the decellularized corneal stromal equivalent *in vivo*

**Figure 7:**
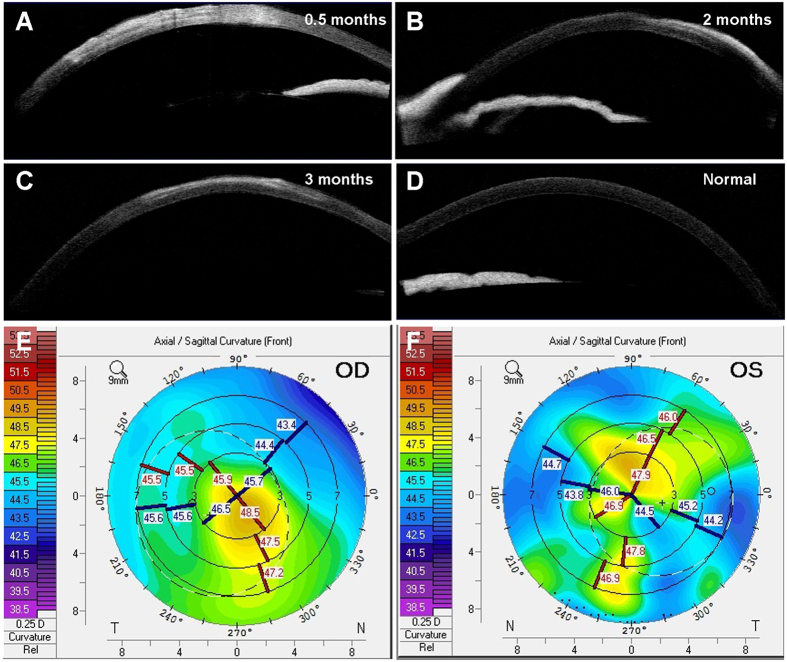
Ophthalmic examination of ALK in rabbits. (**A–C**) AS-OCT revealed corneal and graft edema at 0.5 months after transplantation, which disappeared afterward, whereas the contour profile of the grafts was easily detected with a slightly higher density in the grafts. (**D**) An AS-OCT image of a normal rabbit cornea. (**E**) Corneal topography 3 months after ALK showing mildly irregular with no sign of a keratoconus cornea or other corneal deformation. (**F**) Corneal topography of the contralateral unoperated eye.

We evaluated the effects of implanting the decellularized corneal stromal equivalent *in vivo*. During the 3-month monitoring period, all animals survived without corneal neovascularization, graft degradation, and corneal rejection. The re-epithelialization time of the grafts was 16 ± 2 days (Fig. 3G–R). Evaluation using AS-OCT revealed corneal and graft edema at 0.5 months after transplantation (Fig. 7A). At 2 and 3 months after transplantation, the contour profile of the grafts was easily detected, with a slightly higher density in the grafts, whereas no edema was observed (Fig. 7B,C). In corneal topography images, the corneal surface curvature of the implanted eye appeared to be mildly irregular, whereas no sign of a keratoconus cornea or other corneal deformation was observed at 3 months after transplantation (Fig. 7E,F). After 3 months, the transparency of the grafts significantly improved and was similar to that of the surrounding normal corneal tissues (Fig. 3N,O).

**Figure 8:**
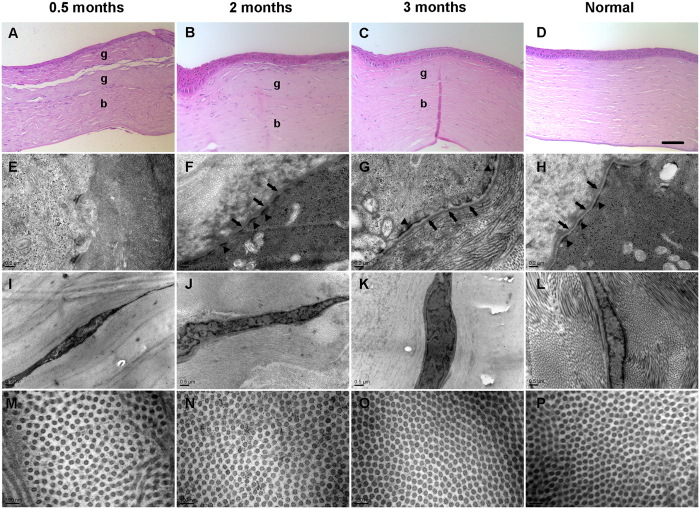
Histological and TEM analysis of ALK in rabbits. (**A–C**) H&E staining depicting the wound healing process of the graft after ALK. (g: graft; b: recipient bed). Bar: 20 μm. (**F–G**) TEM image depicting hemi-desmosomes (arrowheads) and newly synthesized basement membrane (arrows) at the epithelial-stromal interface. (**I–K**) TEM image demonstrating that keratocytes were activated at 0.5 and 2 months after ALK and reverted to the quiescent state at 3 months after ALK. (**M–O**) TEM image revealing that the collagen fibril spacing decreased and was arranged with a uniform spacing, similar to the normal cornea at 3 months after ALK. (**D,H,L,P**) Histological and TEM analysis of a normal rabbit cornea.

Histological analysis was performed to evaluate the wound healing process of the grafts (Fig. 8A–C). At 0.5 months after transplantation, 2–3 layers of corneal epithelial cells covered the grafts, and some keratocytes migrated into the grafts (Fig. 8A). A clear distinction between transplanted grafts and host corneas was observed, and the fibrin glue was dissolved, leaving gaps between two lenticules (Fig. 8A). By 2 months, stratified corneal epithelial cells covered the grafts similar to that of the normal cornea, and the gaps between two lenticules disappeared (Fig. 8B). By 3 months, the corneal epithelial cells of grafts still possessed a stratified layer, and the grafts were almost completely inoculated into the host cornea (Fig. 8C). By TEM, 0.5 months after transplantation, the hemi-desmosome or basement membrane was not observed at the epithelial-stromal interface, whereas keratocytes were activated and the collagen fibril spacing was greater (Fig. 8E,I,M). By 2 months, newly synthesized basement membrane and hemi-desmosomes were observed, although the basement membrane appeared in patches (Fig. 8F). The collagen fibril spacing decreased, but keratocytes remained in an active state (Fig. 8J,N). By 3 months, obvious basement membrane and hemi-desmosomes were observed (Fig. 8G). Collagen fibrils were arranged with a uniform spacing similar to that in the normal cornea, and keratocytes appeared to revert to the quiescent state (Fig. 8K,O).

## Discussion

In this study, we demonstrated the feasibility of constructing a corneal stromal equivalent with SMILE-derived lenticules and fibrin glue. To the best of our knowledge, this is the first histological, ultrastructural, and immunofluorescent analysis of a corneal stromal equivalent with SMILE-derived lenticules and fibrin glue *in vitro* and *in vivo.*

Although corneal transplantation is the best technique for treating deep corneal defects and corneal perforations, it often can’t be performed due to a lack of human donor corneas^1^,^24^. Furthermore, inflammatory and/or immune-mediated conditions often induce a high risk of complications or graft failure after corneal transplantation^10^. Therefore, efforts have been made to identify a practical, accessible, and low biological risk corneal substitute for treating deep corneal defects and corneal perforations^2^,^4^,^17^,^18^,^25^,^26^. Chen *et al*. demonstrated that the fish scale-derived collagen matrix, BioCornea, was capable of sealing full-thickness corneal perforations in pre-clinical mini-pig models^26^. Recently, the acellular porcine corneal stroma (APCS) has been successfully used for lamellar keratoplasty (LKP) in patients with fungal corneal ulcers^25^. However, as a xenogenic graft, APCS has a theoretical risk of zoonotic infection and may lead to potential immune rejection in human hosts^19^,^26^,^27^,^28^,^29^. When corneal defects or corneal perforations are not too large, transplantation of SMILE-derived lenticules with or without fibrin glue is a promising alternative, which can be used temporarily for central corneal defects or corneal perforations (for future optical penetrating keratoplasty) or permanently to repair peripheral corneal defects or corneal perforations^17^,^18^.

As an increasing number of surgeons are inclined to use SMILE to correct myopia, owing to its excellent efficacy, safety, and predictability, an ample amount of lenticule tissue as the immediate by-product of SMILE could be obtained^12^,^30^,^31^,^32^. This feature may reduce the cost of fabrication and provide a sufficient resource for clinical transplantation. Moreover, the corneal stromal equivalent with SMILE-derived lenticules and fibrin glue is easy to prepare and can be kept in glycerol, which is readily available for emergency conditions. To replace the corneal stroma, a construct of sufficient thickness is needed. Given the minor thickness and limited toughness of a single piece of lenticule, 2 or more lenticules could be adhered to each other by fibrin glue to form a corneal stromal equivalent as needed, thus providing a secure suture and reducing the operative time.

As a stromal replacement, the corneal stromal equivalent should facilitate regrowth and adhesion of stable and healthy corneal epithelium^33^. Our *in vitro* studies revealed that primary human corneal epithelial cells seeded on the corneal stromal equivalent formed a well-differentiated epithelium, as shown by the expression of CK12. In addition, basal epithelial cells appeared to maintain a “limbal stem cell phenotype”, which was evident by the expression of ABCG2 and p63α. Desmosomes, hemi-desmosomes, and the underlying newly synthesized basement membrane were also observed. Desmosomes are involved in cell-cell junctions and communication between epithelial cells, whereas hemi-desmosomes and basement membrane are the most mature and steady connections between epithelial cells and corneal stroma^21^,^33^,^34^,^35^. Recent studies have highlighted that corneal epithelial proliferation, differentiation, and wound healing are highly dependent on the support from the underlying stroma, including the extracellular matrix (ECM) and soluble cytokines secreted by keratocytes^36^. In the present study, SMILE-derived lenticules that incorporate viable stromal cells inside may provide a suitable microenvironment for the proliferation and differentiation of corneal epithelial cells *in vitro*.

Corneal graft re-epithelialization after corneal transplantation usually takes no longer than 1 week, whereas the re-epithelialization time of the grafts was 16 ± 2 days in our study^37^,^38^. The delay in re-epithelialization may be attributed to the lack of basement membrane in our corneal stromal equivalent. The basement membrane provides structural support and regulates corneal epithelial cells migration, proliferation, differentiation, adhesion, and apoptosis through various receptors, whereas hemi-desmosomes present on the basal-cell surfaces serve to attach the basal epithelial cells to the basement membrane^21^,^39^. However, the newly synthesized basement membrane and hemi-desmosomes were noted at 2 months after transplantation. In addition, rabbit keratocytes rapidly migrated into the corneal stromal equivalent within 0.5 months. These findings demonstrated that the corneal stromal equivalent is a suitable scaffold that supports the growth of corneal cells.

Given that the human cornea lenticule is a xenogenic graft in rabbits, we used the process of human cornea decellularization previously described by Shafiq *et al*. to create corneal scaffolds from lenticules that preserve the native ECM of the corneal stroma and support the growth of corneal epithelial cells and stromal fibroblasts^19^. Yam *et al*. reported that decellularization of lenticules by sodium dodecylsulfate (SDS) was superior to other treatments, which resulted in minimum ECM disruption^40^. However, Shafiq *et al*. reported that this treatment may not support epithelial growth due to poor attachment of the cells^19^. As complete graft re-epithelialization is critical for graft survival and protection of the stroma against infection and melting, further characterization is necessary to confirm its effect on corneal graft re-epithelialization^38^.

Although it was reconstructed by decellularized lenticules and fibrin glue, the corneal stromal equivalent could gradually become transparent with no corneal neovascularization, rejection reactions, or graft degradation. Consistent with previous studies in which APCS was used, the transparency of the grafts at 3 months after transplantation significantly improved and was similar to that of the surrounding normal corneal tissues^25^. During follow-up, signs of infiltration of neutrophilic leukocytes or leukomonocytes were not observed. Fibrin glue is a tissue adhesive that has been extensively used in lamellar keratoplasty and other ocular surface reconstructive procedures^1^,^41^,^42^,^43^. In addition, fibrin matrices have been proposed as stromal substitutes in different tissues, including corneas^20^. In the present study, we used fibrin glue to keep the corneal stromal equivalent and the recipient attached. Although the fibrin glue spontaneously dissolved within 0.5 months, leaving gaps between two lenticules, the corneal stromal equivalent was integrated with the host cornea and sufficiently supported cornea reconstruction within 2 months. We hypothesized that the endothelial fluid pump pumped fluid out of the cornea, dehydrated the stroma and created a swelling pressure, which held the cornea together^43^. Then, the gap between two lenticules or between a lenticule and the recipient bed was effectively repaired by keratocytes from the rabbit host cornea.

Although the constructed corneal stromal equivalent possessed favourable optical clarity *in vitro* and *in vivo* and the toughness necessary to withstand surgical procedures, further work is required to evaluate its biomechanical properties and light transmittance. In addition, the corneal stromal equivalent used in anterior lamellar keratoplasty was derived from decellularized human lenticules. Implantation of the corneal stromal equivalent with fresh rabbit-derived lenticules that incorporate viable stromal cells and fibrin glue should be further assessed in future investigations.

In conclusion, we demonstrated that the corneal stromal equivalent with SMILE-derived lenticules and fibrin glue could safely, reliably, and effectively repair damage to the anterior cornea, which may provide new avenues in the treatment of deep corneal defects or corneal perforations.

## Additional Information

**How to cite this article**: Yin, H. *et al*. Construction of a Corneal Stromal Equivalent with SMILE-Derived Lenticules and Fibrin Glue. *Sci. Rep.* **6**, 33848; doi: 10.1038/srep33848 (2016).
